# A Diagnostic Approach to Separate Acute Human Bocavirus 1 Respiratory Tract Infection From Long-lasting Virus Shedding

**DOI:** 10.1093/infdis/jiaf130

**Published:** 2025-03-13

**Authors:** Rajita Rayamajhi Thapa, Cristiana Nascimento-Carvalho, Tobias Allander, Tuomas Jartti, Maria Söderlund-Venermo

**Affiliations:** Department of Virology, University of Helsinki, Finland; Department of Pediatrics, School of Medicine, Federal University of Bahia, Salvador, Brazil; Department of Microbiology, Tumor and Cell Biology, Karolinska Institutet, and Department of Clinical Microbiology, Karolinska University Hospital, Stockholm, Sweden; Department of Pediatrics and Adolescent Medicine, Turku University Hospital and University of Turku, Finland; Department of Virology, University of Helsinki, Finland

**Keywords:** diagnostics, endonuclease treatment, ePCR, human bocavirus 1, parvovirus

## Abstract

Human bocavirus 1 (HBoV1) causes mild to life-threatening respiratory tract infections in children but may persist in the airways for months. Currently used polymerase chain reaction may thus lead to false diagnoses and irrelevant codetections. Our aim was to differentiate acute infections from persistent shedding by pretreatment of airway or serum samples with an endonuclease, followed by polymerase chain reaction (ePCR). We show that HBoV1 DNA is protected by a capsid in the acute phase but not in persistent shedding, and we provide proof of concept of a novel test that may be applied in routine diagnosis of acute HBoV1 respiratory tract infection for more accurate results.

## Teaser

We show that human bocavirus 1 DNA is encapsidated in the acute phase but not in persistent shedding, and we introduce a novel test—endonuclease polymerase chain reaction—that can distinguish between these scenarios for a more accurate diagnosis of acute human bocavirus 1 respiratory tract infection.

Human bocaviruses (HBoVs) are single-stranded DNA viruses of the *Parvoviridae* family, genus *Bocaparvovirus* [1]. HBoV1 was discovered in pediatric respiratory samples in 2005 and is the second human-pathogenic parvovirus known [2]. Globally, 2% to 20% of pediatric respiratory tract infections (RTIs) are due to HBoV1, presenting it as one of the most common respiratory viruses [1, 2]. Children aged 0.5 to 5 years are the typical targets, but more rarely HBoV1 may also affect adults [3, 4].

HBoV1 causes typically mild but sometimes even life-threatening pediatric RTIs [1, 5]. The clinical signs and symptoms of HBoV1 infection include cough, rhinitis, fever, dyspnea, and wheezing, often coupled with diarrhea or acute otitis media. Over 25% of children with bronchiolitis have been shown to have acute HBoV1 infection [6]. Also, asthma exacerbations and encephalitis have been observed in sole HBoV1 infections [1]. Apart from HBoV1, 3 related bocaviruses have been discovered (HBoV2–HBoV4), but they are regarded enteric [7].

Approximately for a week after HBoV1 infection (Supplementary Figure 1), high loads of viral DNA (approximately 10^12^ copies/mL) can be detected in the respiratory tract, with a rapid decline in a few days to persist at lower viral loads (<10^4^ copies/mL) for several weeks, months, or even a year [1, 6, 8, 9]. The long persistence of HBoV1 in the airways complicates the interpretation of positive polymerase chain reaction (PCR) results, leading to misrepresentation of the true diagnosis. Hence, PCR alone is insufficient for accurate diagnosis of acute HBoV1 infection. Serology or detection of viral mRNA, viral antigen, or high viral loads in the airways, or viremia, should therefore be used as more trustworthy diagnostic markers for acute primary HBoV1 infection [1, 5, 6, 9–13], but due to technical or sampling difficulties, they are not included in routine diagnostic panels.

We hypothesized that viral genomes detected in the shedding phase are not virion borne but rather noninfective free DNA released from damaged airway tissues. Based on this hypothesis, our study aimed to differentiate acute-phase HBoV1 virions from the unprotected free HBoV1 DNA by endonuclease pretreatment followed by PCR (ePCR), which could be used in routine diagnostics to differentiate acute HBoV1 infections from persistent shedding.

## MATERIALS AND METHODS

### Patients and Samples

For the ePCR, 3 types of samples were analyzed from children with RTI: nasopharyngeal aspirate (NPA), nasopharyngeal swab (NPS), and serum samples. All children had been previously characterized for different stages of HBoV1 infection: acute-phase HBoV1 RTI was evidenced by high viral load (≥10^4–6^ copies/mL, depending on the study) or mRNA in airway samples or IgM, low IgG avidity, or seroconversion (or ≥4-fold IgG increase) in paired sera, whereas nonacute infections had low viral load, no IgM, and high IgG avidity or stable IgG (Supplementary Tables 1–3) [9–14].

We obtained NPA samples from 35 children aged 7 to 48 months with community-acquired pneumonia (Supplementary Table 1A): 22 had acute HBoV1 infection whereas 13 had nonacute infections but were NPA PCR positive [12]. Samples were collected during 2006 to 2011 at the Federal University of Bahia Hospital, Salvador, Brazil. We included 10 deidentified NPA samples shared by the national reference laboratory for respiratory viruses at the Karolinska University Hospital in Sweden: 5 each with high- and low-load HBoV1 by an Allplex respiratory panel (Seegene; Supplementary Table 1B). Furthermore, 16 NPS samples, with follow-up samples taken at 2 weeks and 2 months, were collected from children aged 7 to 23 months with acute wheezing (VINKU2); on admission, 15 of 16 children had acute HBoV1 infection while 1 exhibited HBoV1 persistence (Supplementary Table 2). The samples were collected during 2007 to 2009 at the Turku University Hospital, Finland [13, 14]. Last, 18 serum samples were available from children aged 0.5 to 12 years with acute wheezing (VINKU1), obtained at the Turku University Hospital between 2000 and 2002 [6, 10]. Six children had acute/recent HBoV1 infection whereas 12 had nonacute HBoV1 infection, as determined by serology, but with airway virus shedding (Supplementary Table 3).

### Ethics

The HBoV1 DNA testing of the samples from Finland was approved by the Ethics Committee of the Hospital District of Southwest Finland and, for those from Brazil, by the Ethics Committee of the Federal University of Bahia and the Brazilian Ethics Committee on Research. All patients gave informed consent. The 10 NPA samples provided by the national reference laboratory in Sweden were deidentified. All ethics guidelines of the hospitals and the Helsinki Declaration were followed in the research conduct.

### Endonuclease PCR

**Figure 1.**
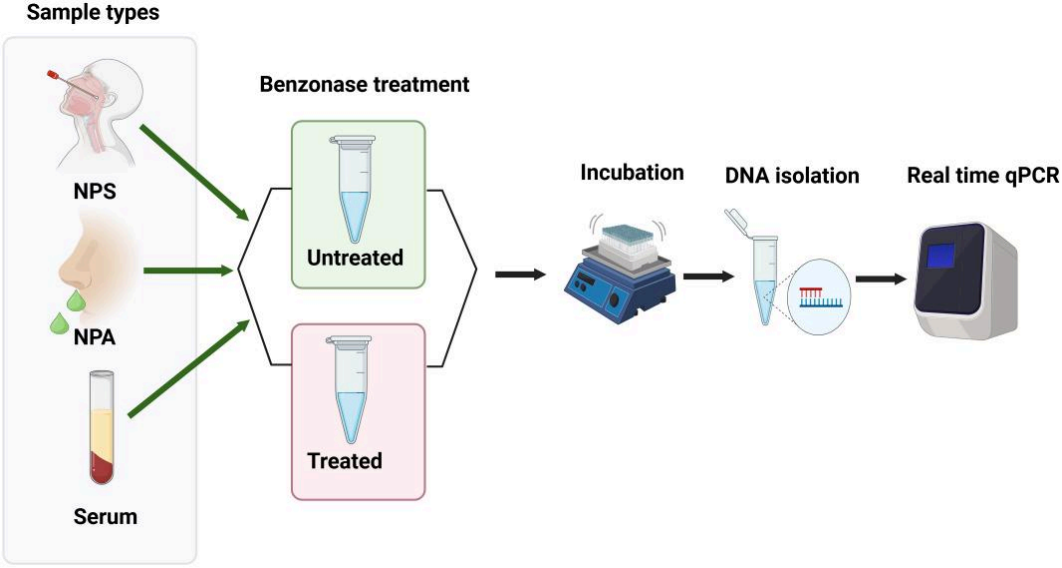
Experimental layout of the endonuclease-based polymerase chain reaction. Created with Biorender https://BioRender.com/k09g841. Abbreviations: NPA, nasopharyngeal aspirate; NPS, nasopharyngeal swab; qPCR, quantitative polymerase chain reaction.

Optimization of the endonuclease pretreatment is shown in the supplement (Supplementary Figure 2). All clinical samples (NPA, NPS, and serum) and controls (HBoV1 plasmid and culture medium virions) were pretreated with Benzonase to investigate whether the HBoV1 DNA was encapsidated (virions) or nonencapsidated (naked DNA), as depicted in Figure 1. For each sample, 100 µL was treated with 2.5-U/µL Benzonase (25 kU; Sigma-Aldrich), and another 100 µL was left untreated; the assay was done in duplicates. Both aliquots were incubated at 37 °C for 60 minutes at 120-rpm shaking, followed by DNA extraction with the QIAamp DNA Mini Kit (Qiagen) and elution with 100-µL kit elution buffer, and stored at −20 °C. HBoV1 singleplex quantitative PCR (qPCR) was performed in duplicates, targeting the left-hand HBoV1 genome as described [11]. In each experiment, the virions and PCR target HBoV1 plasmid served as acute- and persistence-phase controls, respectively, whereas water served as the negative qPCR control.

### Statistical Analysis

Statistical analyses were performed with Prism version 10.1.2 (GraphPad Software). An unpaired *t* test was used for the comparison of Benzonase-treated and untreated clinical and control samples. *P* values <.05 were considered statistically significant.

## RESULTS

**Figure 2.**
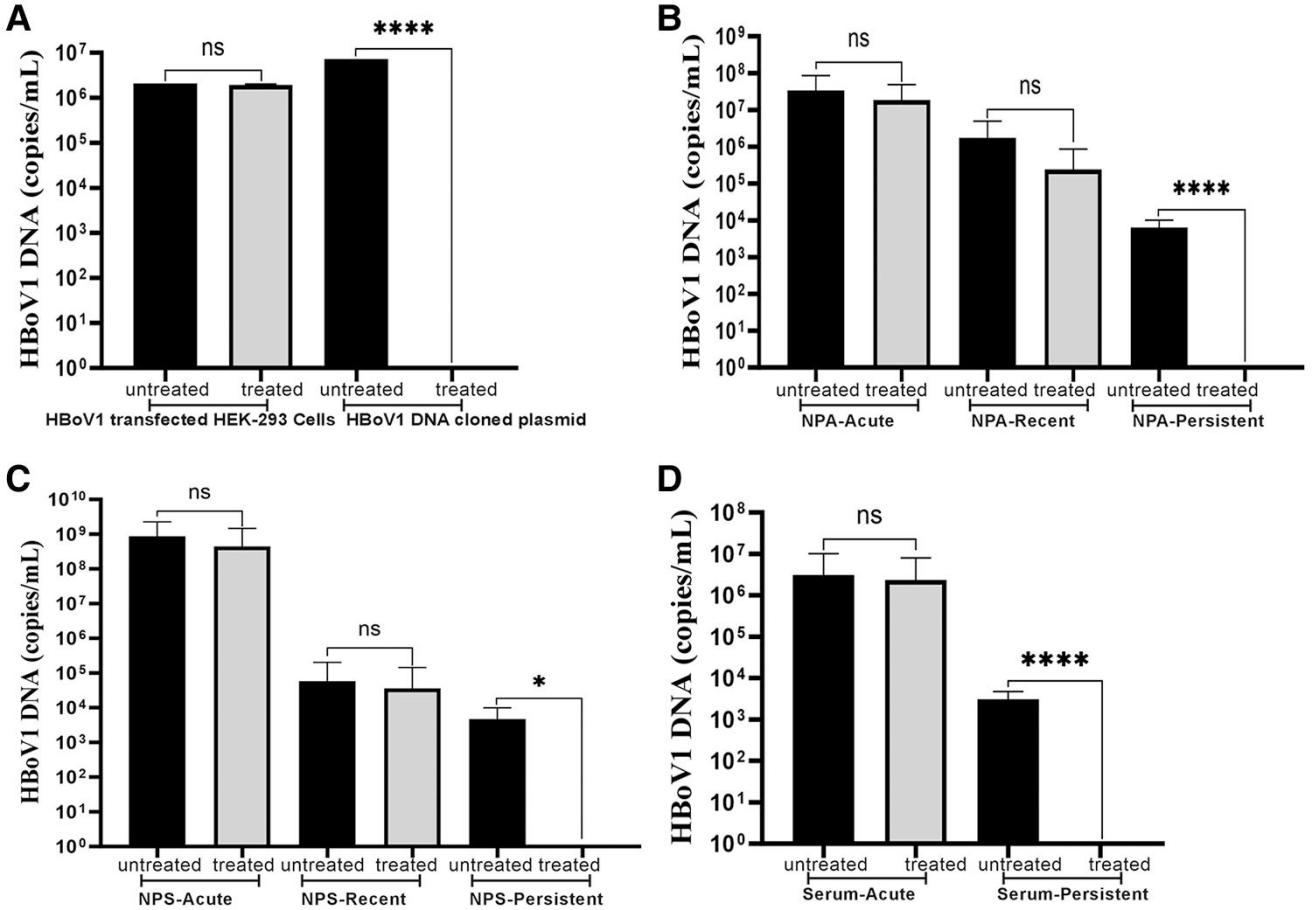
Quantitative PCR of endonuclease pretreated control (*A*) or clinical (*B–D*) samples with 2.5 U/µL of Benzonase, with untreated samples for comparison. *A*, HBoV1 virions from the medium of plasmid HBoV-transfected HEK-293 cell culture and PCR target HBoV1 plasmid as free DNA (supplement). *B*, NPA from acute (n = 7), recent (n = 15), and persistent (n = 13) infections from Brazilian children with community-acquired pneumonia. *C*, NPS from acute (n = 15), recent (n = 12), and persistent (n = 9) infections from children with acute wheezing (VINKU2), Turku, Finland. *D*, Serum samples from acute (n = 6) and persistent (n = 12) infections from children with acute expiratory wheezing (VINKU1), Turku, Finland. All patient samples were previously characterized as being from acute-, recent-, or persistent-phase HBoV1 infection. Results of all the clinical samples are seen in Supplementary Tables 1, 2, and 3. *P < .01. ****P ≤ .0001. HBoV1, human bocavirus 1; NPA, nasopharyngeal aspirate; NPS, nasopharyngeal swab; ns, nonsignificant (*P* > .05); PCR, polymerase chain reaction.

In ePCR, there was no significant difference in HBoV1 DNA copy numbers between Benzonase-treated and untreated HBoV1 virions, whereas the HBoV1 plasmid was reduced to undetectable levels, confirming the degradation of the naked viral DNA and the protection by the capsid (Figure 2*A* and supplement).

Forty-five NPA, 37 NPS, and 18 serum samples were obtained from 79 children with different stages of HBoV1 infection. In the ePCR of all 3 sample types from acute HBoV1 infection, the viral DNA remained detectable, whereas the HBoV1 DNA in samples from all past infections became undetectable (Supplementary Tables 1–3, Figure 2). Among the NPS collected at 2 weeks, 8 samples had in the ePCR partial ≤2-log reductions, implying that the samples contained both HBoV1 virions and naked DNA, while in 4 samples the HBoV1 DNA became undetectable (Supplementary Table 2). Correspondingly, all NPS samples collected at 60 days were sensitive to endonuclease treatment, indicating that the samples contained unprotected HBoV1 DNA. Also, among the serum samples, there were acute-phase samples exhibiting ≤2-log reductions of viral DNA, which may contain both HBoV1 virions and naked DNA (Supplementary Table 3). Results of ePCR of NPA, NPS, and serum samples are shown in Figure 2. All controls worked as expected in all experiments.

## DISCUSSION

This study provides proof of concept of a new approach of combining endonuclease pretreatment and PCR (ePCR) to differentiate HBoV1 acute infection from the notorious prolonged shedding of HBoV1. In the ePCR, 2.5-U/µL endonuclease could degrade an HBoV1 DNA–containing plasmid, even at approximately 10^7^ copies/mL, to an undetectable level, whereas the same amount of infective virions showed no significant reduction in DNA loads. This clearly revealed that naked DNA was totally degraded and that the capsid could protect the genome from degradation. Similar capsid protection has been shown for parvovirus B19 in blood, disclosing that the B19V PCR–positive blood in some circumstances contained naked viral genomes [15]. Hence, our hypothesis was that in acute-phase HBoV1 RTI, the DNA is inside protective capsids as virions, whereas the notorious prolonged virus shedding comprises noninfective naked HBoV DNA. Routine diagnosis of virus infections in RTI is generally made by nonquantitative PCRs in airway samples, which cannot differentiate between acute HBoV1 infection and persistent virus shedding, thereby displaying clinically false diagnoses and coinfections.

We assessed different clinical sample types (NPA, NPS, and serum) collected from children with acute RTI with HBoV1 PCR–positive airway samples but with different stages of HBoV1 infection previously characterized by other methods [6, 9–14]. The results obtained in our ePCR demonstrated that HBoV1 DNA indeed was protected inside capsids, as virions, in all samples taken at the acute phase. In the follow-up NPS samples taken 2 weeks postadmission, two-thirds of the available samples still contained some protected (encapsidated) viral DNA, whereas in samples taken at 2 months, all viral DNA was fully degraded. Likewise, for all the samples from children characterized with past HBoV1 infection, the viral DNA was degraded upon endonuclease pretreatment, indicating that the HBoV1 genomes were unprotected, which suggested that the true etiology for these RTI episodes was not HBoV1. A third of the Brazilian children had 3 to 6 codetected viruses in NPA, with half of them exhibiting acute HBoV1 infection. HBoV1 may thus not be the only virus with prolonged shedding where our ePCR method could be applied.

We did not know the exact stage of RTI at sampling, but the disappearance of HBoV1 IgM (Supplementary Figure 1) crudely correlates with the transition from virions to naked DNA. IgM, despite being an acute-phase marker, may persist for months. Hence, we used other acute-phase markers, such as mRNA and diagnostic IgG data (4-fold increase, seroconversion, or low avidity) for more accurate staging of HBoV1 infection [6, 9–14]. Some of the acute- or recent-phase samples had partial 2-log reductions after the endonuclease treatment. This could be due to individual variations or that children come for sampling at different stages of infection. The time of sampling plays a critical role in the interpretation of test results: some children will be sampled after the first signs of illness, whereas others may wait for a week before seeking health care. However, almost all the children in this study already had IgM on admission, but not all had IgG, suggesting an average time of approximately 1 week from onset of infection to seeking medical care. Nevertheless, 3 of the children providing serum for this study showed seroconversion for IgM, as previously reported [6]. At later stages of infection, it became clear that the HBoV1 DNA was no longer from active replication and no longer infective. This is in line with the short detection period of mRNA and antigen [1, 10, 13]. The rapid decline of HBoV1 virions is likely due to efficient neutralization of HBoV1 by the immune response, which corresponds to recovery from the disease.

Because PCR detects even small amounts of HBoV1 DNA, it does not discriminate acute infection from persistent shedding. Endonuclease pretreatment could be included as an additional approach to attest the stage of infection. After acute HBoV1 infection, the overall levels of HBoV1 DNA decreases over time and may remain relatively stable at around 10^3–4^ copies/mL, eventually to decline below the detection limit during a few weeks to even a year [1, 8]. Our ePCR results correlate well with viral loads and detection of HBoV1 mRNA (Supplementary Figure 1). However, the viral load in airway samples is not always a reliable indicator of acuteness [6]. HBoV1 DNA becomes increasingly sensitive to endonuclease with time after acute HBoV1 infection, so ePCR could provide better evidence of acuteness than qPCR, without loosing sensitivity. Despite the benefits, there are some drawbacks in hospital-based diagnostics: the additional cost of Benzonase, inclusion of an additional step in sample treatment, and the need of parallel tubes. Furthermore, we used freeze-thawed samples. It remains to be investigated whether an additional cell lysis step is necessary for fresh samples—yet, ePCR did work well for cell-free serum. Nevertheless, such accurate diagnostics is generally needed only in severe infections and epidemiologic studies [1, 5, 6].

In conclusion, our results provide proof of concept that long-term HBoV1 DNA shedding mainly consists of nonencapsidated viral DNA and that endonuclease treatment prior to PCR (ePCR) can differentiate between acute HBoV1 RTI and persistent shedding. Hence, ePCR of NPA and NPS samples has potential use in the routine diagnosis of HBoV1 infections without the need for qPCR, reverse transcription PCR, or serology. We further showed that this assay can be applied to serum. Additionally, the sensitivity of the assay is equal to that of the PCR already in use in hospital laboratories but without the need to worry about virus shedding or false coinfections.

## Supplementary Material

jiaf130_Supplementary_Data

## Supplementary Data

Supplementary materials are available at *The Journal of Infectious Diseases* online (http://jid.oxfordjournals.org/). Supplementary materials consist of data provided by the author that are published to benefit the reader. The posted materials are not copyedited. The contents of all supplementary data are the sole responsibility of the authors. Questions or messages regarding errors should be addressed to the author.

## Notes

***Acknowledgments.*** We thank Jianming Qiu for the full-length HBoV plasmid clone that was used to transfect HEK cells in our previous study [13].

***Author contributions*.** Investigation, methodology, analysis: R. R. T. Writing of original draft and review and editing: R. R. T. and M. S.-V. Funding acquisition: R. R. T. and M. S.-V. Resources: C. N.-C., T. A., T. J., and M. S.-V. Conceptualization, methodology, project administration, and supervision: M. S.-V. Review and editing: R. R. T., C. N.-C., T. A., T. J., and M. S.-V.

***Data availability*.** Data are available in the supplementary material.

***Financial support***. This work was supported by the Doctoral Programme in Population Health (to R. R. T.); the Sigrid Jusélius Foundation (M. S.-V.); and the Life and Health Medical Association (M. S.-V.).
