# Supplementary material for: A Diagnostic Approach to Separate Acute Human Bocavirus 1 Respiratory Tract Infection From Long-lasting Virus Shedding
Source: J Infect Dis. 2025 Mar 13;231(5):e862–6. doi: 10.1093/infdis/jiaf130 (PMC12128058; doi:10.1093/infdis/jiaf130)
Supplement: jiaf130_Supplementary_Data [file jiaf130_supplementary_data.pdf]

SUPPLEMENT

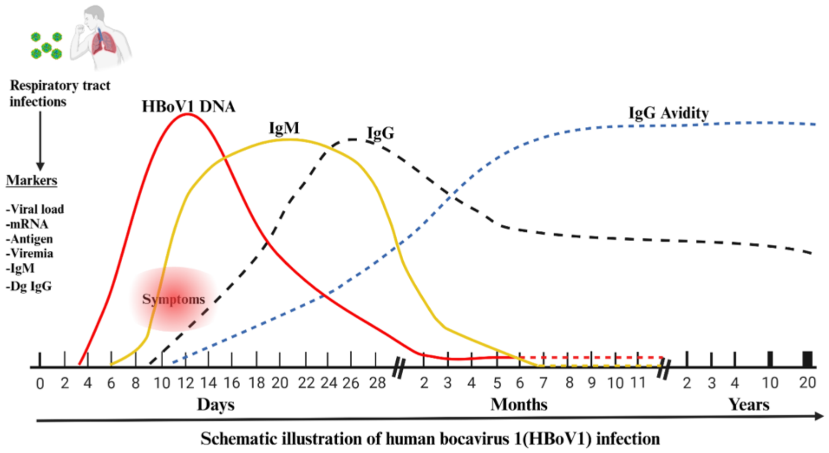

Figure S1. Schematic overview of HBoV1 infection. High viral load and presence of mRNA in the airways, and positive IgM, low IgG avidity, and seroconversion, are the markers of acute HBoV1 infection. The exact time of infection plays a crucial role in the diagnosis of acute infection. NPA, nasopharyngeal aspirates; NPS, nasopharyngeal swab; IgM, immunoglobulin M; IgG, immunoglobulin G; Dg IgG, diagnostic IgG (low IgG avidity, seroconversion or four-fold increase of IgG in paired sera) (1).

Optimisation of endonuclease pretreatment

Benzonase (25 kU; Sigma-Aldrich) is a genetically engineered endonuclease, obtained from *Serratia marcescens,* which is known to degrade all free genetic material in the sample (2). To determine its efficiency in degrading HBoV1 DNA, a plasmid (pHBoV1) containing the PCR-target region (UTR-NS1) of the HBoV1 genome with 10^7 copies/mL, was pretreated in triplicates with 0, 0.5, 1, 1.5, 2, and 2.5 Units (U)/µl of Benzonase at 37°C for 60 minutes with 120 rpm shaking. To show that capsids protect the viral genomes, HBoV1 virions from the medium of previously transfected human embryonic kidney (HEK-293) cells (3,4), underwent the same Benzonase treatment with 0 and 2.5 U/µl. Both the pretreated pHBoV1 and virions then underwent DNA extraction and qPCR.

As shown in **Fig. S2**, the amount of naked pHBoV1 DNA was significantly reduced in the different concentrations of Benzonase compared to the untreated ($9x10^6$ copies/mL) control, reaching total degradation at 2.5 U/µL. As shown in **Fig. 2A**, with 2.5 U/µL endonuclease, there was no significant difference in HBoV1 DNA copy numbers (at $2x10^6$ copies/mL) between Benzonase treated and untreated HBoV1 virions, whereas pHBoV1 (at $7x10^6$ copies/mL) was reduced (100%) to undetectable levels, confirming the degradation of the naked viral DNA (Fig 1B). Hence, 2.5 U/µL of Benzonase was selected for the ePCR assay of the clinical samples.

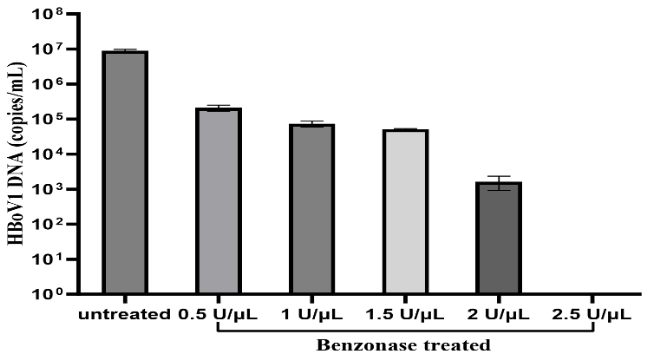

Figure S2. Optimization of Benzonase concentration using a plasmid with the target genome region of HBoV1 as naked DNA. All done in triplicate. U/µl: Units/microliter.

Table S1. Human NPA samples, untreated and treated with endonuclease to differentiate acute HBoV1 infection from persistent shedding in Brazilian children with community-acquired pneumonia CAP (A) and Swedish children with respiratory tract infections (B).
A)

NPA ID	Untreated-copies/mL HBoV1 load	Treated - copies/mL HBoV1 load	Phase of infection	Previous HBoV1 serology*	qPCR positive for the following viruses
1	1.30E+07	7.90E+06	acute	IgM+, IgG+ increase	AdV, MPV, HBoV1, HEV
2	2.30E+06	1.30E+05	recent	IgM+, IgG+	HBoV1, RSVA, HRV, HEV
3	1.40E+06	3.90E+04	recent	IgM+, IgG+	HBoV1, RSVA
4	4.20E+05	3.50E+04	recent	IgM+, IgG+	AdV, HBoV1, RSVA, HEV
5	1.80E+06	1.00E+05	recent	IgM+, IgG+	HBoV1, PIV3
6	3.70E+05	3.00E+05	acute	IgM+, IgG+ increase	AdV, HBoV1, RSVA, PIV3
7	7.90E+05	1.40E+04	acute	IgM+, IgG+ increase	HBoV1, RSVA,
8	1.10E+05	1.40E+04	recent	IgM+, IgG+	AdV, HBoV1, HRV, HEV
9	9.90E+05	4.70E+04	recent	IgM+, IgG+	ADV, HBoV1
10	1.30E+07	6.30E+05	recent	IgM+, IgG+	HBoV1
11	1.20E+05	2.40E+04	recent	IgM+, IgG+	HRV, HEV, HBoV1
12	1.50E+05	1.00E+04	acute	IgM+, IgG seroconversion	HBoV1
13	8.10E+05	3.40E+04	recent	IgM+, IgG+	HBoV1
14	6.20E+05	3.30E+04	recent	IgM+, IgG+	HBoV1
15	6.30E+05	4.30E+04	recent	IgM+, IgG+	HBoV1
16	3.70E+05	1.10E+04	recent	IgM+, IgG+	HBoV1
17	1.10E+05	7.00E+04	recent	IgM+, IgG+	HBoV1
18	1.20E+08	6.90E+07	acute	IgM+, IgG+ increase	HBoV1
19	1.00E+08	5.40E+07	acute	IgM+, IgG+ increase	HBoV1
20	1.10E+05	2.40E+03	recent	IgM+, IgG+	HBoV1
21	3.20E+05	3.00E+04	acute	IgM+, IgG+ increase	HBoV1
22	2.80E+06	2.40E+06	recent	IgM+, IgG+	HBoV1
Mean	1.18E+07				
Median	7.10E+05				
23	1.03E+04	0.00E+00	past	IgG+ stable, IgM neg	AdV, HBoV1, INFA, HRV, RSVB, HEV
24	1.11E+04	0.00E+00	past	IgG+ stable, IgM neg	HBoV1, RSVA, HEV
25	7.25E+03	0.00E+00	past	IgG+ stable, IgM neg	AdV, MPV, HBoV1, HRV, PIV1, PIV3
26	3.40E+03	0.00E+00	past	IgG+ stable, IgM neg	AdV, MPV, HBoV1, HEV
27	3.95E+03	0.00E+00	past	IgG+ stable, IgM neg	HBoV1, HRV
28	9.62E+03	0.00E+00	past	IgG+ stable, IgM neg	HBoV1, HEV, PIV3
29	1.21E+04	0.00E+00	past	IgG+ stable, IgM neg	HBoV1, HRV, OC43
30	3.82E+03	0,00E+00	past	IgG+ stable, IgM neg	HBoV1
31	1.01E+04	0.00E+00	past	IgG+ stable, IgM neg	HBoV1
32	3.54E+03	0.00E+00	past	IgG+ stable, IgM neg	HBoV1
33	2.72E+03	0.00E+00	past	IgG+ stable, IgM neg	HBoV1
34	2.49E+03	0.00E+00	past	IgG+ stable, IgM neg	HBoV1
35	1.96E+03	0.00E+00	past	IgG+ stable, IgM neg	HBoV1
Mean	6.33E+03				
Median	3.95E+03				

B)

NPA ID	Untreated- copies/mL HBoV1 load	Treated - copies/mL HBoV1 load	Phase of infection**	Allplex PCR Ct	Other virus detections (Allplex)
1	1.23E+08	1.06E+08	NA	18.9	
2	4.51E+08	2.18E+07	NA	16.4	
3	1.14E+08	6.32E+07	NA	19.9	
4	3.23E+05	1.37E+05	NA	26.8	PIV3
5	3.65E+07	6.33E+06	NA	21.1	CoV-NL63, RV
6	2.13E+06	5.89E+04	NA	38.0	OC43/HKU1, RV
Mean	1.21E+08				
Median	7.54E+07				
7	5.11E+03	0.00E+00	NA	38.8	RV
8	1.45E+04	0.00E+00	NA	34.4	
9	1.58E+03	0.00E+00	NA	40.0	
10	1.51E+04	0.00E+00	NA	38.6	RV
Mean	9.09E+03				
Median	9.82E+03				

*The staging of infection has been published before based on serology results of acute and convalescent sera (5). **For these routine NPA samples no other diagnostic tests have been done, so the staging of infection relies on the qPCR, with low Ct (<30) values indicating high viral loads, supporting an acute phase of infection and high Ct (≥30) values indicating low viral loads, supporting persistent shedding. Sample #6 may be a mix of both virions and naked DNA exhibiting a 2-log difference in viral load, suggesting a borderline result, i.e., a sub-acute or recent infection. NA, not available; increase, increase in IgG in paired serum samples.

Table S2. Human HBoV1 PCR-positive NPS samples (on admission, with follow-up samples), from Finnish children with acute wheezing, untreated and treated with endonuclease to differentiate acute HBoV1 infection from persistent shedding.

NPS ID	On admission				At two weeks post admission		At two months post admission	
	Untreated- copies/mL HBoV1 load	Treated - copies/mL HBoV1 load	Previous results*	Phase of infection	Untreated- copies/mL HBoV1 load	Treated - copies/mL HBoV1 load	Untreated- copies/mL HBoV1 load	Treated - copies/mL HBoV1 load
1	1.58E+07	4.09E+06	IgM+, dg IgG, mRNA+	acute	NA	NA	NA	NA
2	6.82E+05	2.84E+05	IgM+, dg IgG, mRNA+	acute	9.46E+03	0	8.17E+02	0
3	5.23E+09	3.54E+09	IgM+, dg IgG, mRNA+	acute	1.34E+03	0	2.39E+03	0
4	3.17E+08	8.83E+07	IgM+, dg IgG, mRNA+	acute	3.17E+03	0	NA	NA
5	6.64E+08	4.77E+07	IgM+, dg IgG, mRNA+	acute	1.19E+05	2.45E+04	6.70E+02	0
6	2.18E+09	1.94E+09	IgM+, dg IgG, mRNA+	acute	5.05E+05	3.78E+05	1.45E+04	0
7	2.49E+07	1.93E+06	IgM+, dg IgG, mRNA+	acute	1.03E+04	8.96E+02	1.97E+03	0
8	6.76E+08	1.11E+08	IgM+, dg IgG, mRNA+	acute	4.08E+03	8.16E+02	1.65E+03	0
9	5.43E+08	4.45E+07	IgM+, dg IgG, mRNA+	acute	1.26E+04	3.02E+03	4.70E+03	0
10	1.18E+06	1.44E+05	IgM+, dg IgG, mRNA+	acute	8.72E+03	2.47E+03	5.12E+03	0
11	8.81E+08	2.52E+08	IgM+, dg IgG, mRNA+	acute	5.68E+03	3.78E+03	NA	NA
12	8.42E+08	1.08E+08	IgM+, dg IgG, mRNA+	acute	6.33E+03	0	1.27E+03	0
14	8.54E+08	1.40E+08	IgM+, dg IgG, mRNA+	acute	1.65E+04	1.33E+04	NA	NA
15	8.51E+06	1.18E+06	IgM+, dg IgG, mRNA+	acute	NA	NA	NA	NA
16	1.38E+04	0	IgM neg, IgG+ stable	past	NA	NA	NA	NA
Mean	8.16E+08							
Median	5.43E+08							

*The staging of infection has been published before (6,7). Dg IgG, diagnostic IgG (increase in titer, seroconversion, or low IgG avidity); NA, not available.

Table S3. Human serum samples, untreated and treated with endonuclease to differentiate acute HBoV1 infection from persistent shedding in children with acute expiratory wheezing.

Serum ID	Untreated - copies/mL HBoV1 load	Treated - copies/mL HBoV1 load	Phase of infection	Previous results*
1	7.01E+05	1.75E+05	acute	IgM+, IgG increase
2	1.74E+07	1.38E+07	acute	IgM+, IgG seroconversion
3	1.26E+05	1.23E+04	acute	IgM+, IgG seroconversion
4	1.40E+05	8.58E+04	acute	IgM+, IgG seroconversion
5	2.34E+05	8.14E+03	acute	IgM+, IgG seroconversion
6	1.42E+05	2.89E+03	acute	IgM+, IgG seroconversion
Mean	3.12E+06			
Median	1.88E+05			
7	5.86E+03	0	past	IgG+ stable, IgM neg
8	5.55E+03	0	past	IgG+ stable, IgM neg
9	1.44E+03	0	past	IgG+ stable, IgM neg
10	2.10E+03	0	past	IgG+ stable, IgM neg
11	3.28E+03	0	past	IgG+ stable, IgM neg
12	2.84E+03	0	past	IgG+ stable, IgM neg
13	1.08E+03	0	past	IgG+ stable, IgM neg
14	2.05E+03	0	past	IgG+ stable, IgM neg
15	3.39E+03	0	past	IgG+ stable, IgM neg
16	2.50E+03	0	past	IgG+ stable, IgM neg
17	5.35E+03	0	past	IgG+ stable, IgM neg
18	1.27E+03	0	past	IgG+ stable, IgM neg
Mean	3.06E+03			
Median	2.67E+03			

* The staging of infection has been published (8,9). All included sera were the first of paired sera (VINKU1 cohort), taken at admission. ID, identification.

References:

1. Söderlund-Venermo M, Qiu J. Human boca- and protoparvoviruses (*Parvoviridae*). In: Encyclopedia of Virology. 4th ed. Elsevier; 2021:426.
2. Pyeon D, Lambert PF, Ahlquist P. Production of infectious human papillomavirus independently of viral replication and epithelial cell differentiation. Proceedings of the National Academy of Sciences. 2005; 9311-6.
3. Huang Q, Deng X, Yan Z, et al. Establishment of a reverse genetics system for studying human bocavirus in human airway epithelia. PLoS Pathog. 2012;8:e1002899.
4. Xu M, Perdomo FM, Mattola S, et al. Persistence of human bocavirus 1 in tonsillar germinal centers and antibody-dependent enhancement of infection. mBio 2021;12:e03132-20.
5. Nascimento-Carvalho AC, Vilas-Boas AL, Fontoura MH, et al. Serologically diagnosed acute human bocavirus 1 infection in childhood community-acquired pneumonia. Pediatr Pulmonol. 2018;53 :88-94.
6. Xu M, Arku B, Jartti T, et al. Comparative diagnosis of human bocavirus 1 respiratory infection with messenger RNA reverse-transcription polymerase chain reaction (PCR), DNA quantitative PCR, and serology. J Infect Dis. 2017;215:1551-1557.
7. Turunen R, Koistinen A, Vuorinen T, et al. The first wheezing episode: respiratory virus etiology, atopic characteristics, and illness severity. Pediatr Allergy Immunol 2014; 25:796–803.
8. Söderlund-Venermo M, Lahtinen A, Jartti T, et al. Clinical assessment and improved diagnosis of bocavirus-induced wheezing in children, Finland. Emer Infect Dis. 2009;15:1423.
9. Kantola K, Hedman L, Arthur J, et al. Seroepidemiology of human bocaviruses 1–4. J Infect Dis. 2011;1403–1412.
